# Transcending the self to transcend suffering

**DOI:** 10.3389/fpsyg.2023.1113965

**Published:** 2023-07-07

**Authors:** Brian H. Ge, Fan Yang

**Affiliations:** Department of Psychology, University of Chicago, Chicago, IL, United States

**Keywords:** self-transcendence, suffering, human existence, meaning in life, coping strategies, positive emotions

## Abstract

Suffering is inevitable in human life. Our perspective paper theorizes on precise mechanisms for how *self-transcendence*—the state in which an individual looks beyond the self and adopts a larger perspective including concern for others and the world—may help people endure the experience of suffering. From an examination of empirical literature ranging from social psychology to clinical research, we propose that self-transcendence may aid the endurance of suffering along three psychological levels: (1) On the level of affect, the unique profundity and positivity of *self-transcendent experiences* (e.g., awe, flow, compassion) may *supersede and reduce the salience* of negative affect arising from suffering (e.g., fear, despair, depressive mood). (2) On the level of cognition, the larger frame of reference provided by *self-transcendent thinking* may *contextualize* one’s suffering as something comprehendible, thereby helping to resolve the challenges of making meaning from suffering (e.g., that one’s existing meaning systems are unable to explain the suffering event). (3) On the level of motivation, the drive to fulfill one’s *need for self-transcendence* may counterbalance the more hedonically-oriented motivations that can promote negative coping strategies in response to suffering (e.g., avoidance, substance abuse). All three mechanisms may also provoke downstream prosocial behaviors that help embed the individual into networks of social support. Altogether, by synthesizing specific mechanisms from affective, cognitive, and motivational self-transcendent processes, our paper establishes a theoretical framework for how self-transcendence may help people endure and transcend suffering, thereby elevating the conditions and experiences of our existence.

## Introduction

The history of human suffering is arguably as ancient as humanity itself. The oldest work of poetry in print today, The Epic of Gilgamesh (ca. 2750-2500 B.C.E./2003), details the story of a mighty king tormented by the grief he feels for his beloved’s death as well as by the terror he suffers at the thought of his own. Whether it surfaces in Gilgamesh’s ancient city of Uruk or in the intensive care unit of a modern hospital, suffering has been considered one of the great tragedies fundamental to human existence (e.g., Frankl, 1946/1992). For certain thinkers of philosophy, suffering in life is often unavoidable and too grueling to bear, so much so that it would have been better to have never come into being (e.g., Benatar, 2008; Schopenhauer, 2020). In spite of such doctrines, humanity has persisted all the same, driven perhaps by the hope that one might still derive from existence some durable reason or means to face one’s suffering and endure it. Substantial empirical research has examined various strategies (e.g., self-regulation of emotion) and factors (e.g., social support) that may help people cope with events of adversity in life, mostly from clinical perspectives and targeted at specific forms of suffering (e.g., trauma, illness, psychological disorders) (e.g., Matthews and Cook, 2009; Freh et al., 2013; Papa et al., 2013; Shear et al., 2014; Gruszczyńska and Knoll, 2015; Shear, 2015). The present perspective paper aims to articulate how *self*-*transcendence—*the state in which an individual looks beyond the self and adopts a larger perspective including concern for others and the world—may help people endure suffering as a form of positive coping. Building on existing literature and consistent with the propositions of second-wave positive psychology (Wong, 2019), we propose a theoretical framework specifying the effects of self-transcendence along three levels of analysis: the affective, the cognitive, and the motivational.

What precisely is “suffering”? A common definitional choice is to conflate it with pain (e.g., Vanden Bos, 2007). Although in everyday language, “pain” and “suffering” are indeed mentioned together frequently and even interchangeably, there are key distinctions between the two. Pain is the direct unpleasant physical or psychological sensation that may arise from different activities or events in life. Pain often can cause suffering, but not necessarily so. Some pain, such as what is felt when eating spicy food, taking a hot bath, or even feeling the muscle-burn of a strenuous workout can be experienced by many people as desirable (Bloom, 2021) instead of as a form of suffering. Other typically more severe cases of physical or psychological pain, such as what is felt when becoming terminally ill or struggling with a psychological disorder (Pompili et al., 2012; Wachholtz et al., 2016; Svenaeus, 2020; Lewis et al., 2021), are more likely to be experienced as negative and aversive. Many philosophers and psychologists consider that it is this mental disruption or aversion in response to pain that qualifies as suffering (e.g., Kauppinen, 2019; McClelland, 2019; VanderWeele, 2019; Brady, 2021; Stilwell et al., 2022). We thus adopt the view that suffering is not the sensation of pain itself, but rather *the negative and aversive mental state in response to physical or psychological pain*. Conceptualizing suffering as a mental state rather than purely a sensation opens the possibility that it can be potentially moderated by psychological mechanisms, the processes of which we will articulate in this paper.

Self-transcendence is a reorientation from egotism toward concern for others and the world. Self-transcendence has been examined from diverse perspectives in the literature, including as a phenomenological experience of ego-dissolution, a set of prosocial motivations and behaviors, a subtype of emotion, an attribute of religious experience, as well as a constellation of character strengths (Montemaggi, 2017; Stellar et al., 2017; Yaden et al., 2017; Kitson et al., 2020; Lavy and Benish-Weisman, 2021; Liu et al., 2022). As a multi-faceted psychological phenomenon, the common feature across different manifestations of self-transcendence is that egotistic interests cease to be the individual’s predominant focus; a perceived higher value presents itself in a target beyond the self, whether in other people, divinity, or even an overarching concept of nature (Stellar et al., 2017; Castelo et al., 2021; Magyar-Russell et al., 2022). Our paper examines the construct of self-transcendence at three levels of psychological processes: affect, cognition, and motivation. We surmise that these three levels may each have distinct benefits for the endurance of suffering and may also interact together in their functions.

The notion that self-transcendence may be a balm for suffering has rich roots in philosophy and psychology, particularly with regards to its theoretical connection to life-meaning. For example, based on his experiences in the Holocaust, Frankl (1946/1992) proposes that the life-meaning which best aids the endurance of suffering is discovered from devotion to something beyond the self. In an effort to solidify Frankl’s propositions into operable constructs, Wong (2013) defines meaning as a multidimensional construct in the PURE model (i.e., purpose, understanding, responsible action, emotional evaluation). The model lays out meaning’s implications for suffering, with self-transcendence referenced as a significant source of such meaning. The benefits of meaning in contending with adversity have been subsequently examined in the literature of meaning-centered coping (e.g., Eisenbeck et al., 2021; Sanchez-Ruiz et al., 2021; Avsec et al., 2022; Eisenbeck et al., 2022), and additional study in other frameworks has likewise affirmed the perceived link between self-transcendence and meaning (Huang and Yang, 2022). We wish to emphasize here, however, that although self-transcendence is closely related to the concept of meaning, it is nonetheless fruitful to examine self-transcendence as a distinct and coherent construct of its own. Our definition of self-transcendence—the state of looking beyond the self toward a larger perspective including others and the world—is concerned fundamentally with how one relates to the self. Such a definition differs from that of meaning, which has been conceptualized as consisting of sub-components like life-coherence, significance, purpose, and experiential appreciation (e.g., Martela and Steger, 2016; Kim et al., 2022). Our intent is specifically to help build upon the qualities and functions of the self-transcendence construct. Where Frankl’s (1946/1992) proposition is that self-transcendence may give rise to a sense of meaning that helps people endure suffering, we wish to focus on self-transcendence in particular and theorize more specifically how it may help people endure suffering—not only through meaning—but through all manner of affective, cognitive, and motivational mechanisms.

On this subject of understanding self-transcendence’s benefits, Wong et al. (2021) frame self-transcendence as an overarching means by which suffering might be integrated into pursuit of perceived higher goals or values. Self-transcendence in this proposition involves unconditional investment in the betterment of others (Communion), the actualizing of a better form of self (Hope), and the reverence of an ideal (Faith). In this manner, the endurance of personal suffering would be made possible *via* dedication to these three domains beyond the self (Wong et al., 2021). Empirical study has affirmed that self-transcendent values and experiences bear positive associations with diminished depressive symptoms, improved emotional well-being, and post-traumatic growth (Bojanowska and Kaczmarek, 2022; Monroy and Keltner, 2022; Xie et al., 2022).

We propose that self-transcendence bears benefits for the endurance of suffering along three psychological levels: (1) On the level of affect, the unique profundity and positivity of *self-transcendent experiences* (e.g., awe, flow, compassion) may *supersede and reduce the salience* of negative emotions arising in suffering (e.g., fear, despair, bitterness). (2) On the level of cognition, the larger frame of reference provided by *self-transcendent thinking* may *contextualize* one’s suffering as something comprehendible, thereby helping to resolve the challenges of making meaning from suffering (e.g., that one’s existing meaning systems are unable to explain the suffering event). (3) On the level of motivation, the drive to fulfill one’s *need for self-transcendence* may counterbalance the more hedonically-oriented motivations that can promote negative coping strategies in response to suffering (e.g., avoidance, substance abuse). Beyond these direct benefits, self-transcendence is theorized also to promote downstream behavioral and social consequences that wield their own benefits, including strengthening one’s connections to networks of social support. We discuss further the mechanisms involved at each level of analysis as well as their interactions below.

## Effects of self-transcendent experiences on suffering

The experience of suffering often entails negative affect, such as depressive mood states or negative emotions like grief, guilt, and anger (Freh et al., 2013; Kim et al., 2021). Severe negative affect may disrupt facets of daily life, as with cases of unexpected rushes of negative emotion accompanying intrusive thoughts (e.g., Calhoun, 2013b; Park and Kennedy, 2017). This attribute of intrusiveness indicates that the negative affect in suffering has potential to metastasize; otherwise-neutral domains of living may, in their disruption by these sudden upsurges, become themselves sources of additional suffering (VanderWeele, 2019). Excessive negative emotion has been connected to the development of depressive disorders, complications in grieving in cases of bereavement, and even detriments to physical health (Keyes, 2002; Zhang et al., 2006; Young et al., 2019; Frumkin et al., 2021). Therefore, the salience and potential intrusiveness of negative affect comprise a predominant problem to be addressed in the endurance of suffering.

Due to its power to promote profoundly positive affective states, self-transcendent experiences may reduce the salience of negative affect in suffering. Self-transcendent experiences (e.g., flow, awe, compassion) are characterized by the perceived dissolution of the boundaries of the self and an enhanced unity with other people or the world (Stellar et al., 2017; Yaden et al., 2017). This sense of unity is associated with a positive—even ecstatic—affective state, in tandem with a diminishing salience of selfhood (Hood, 1975; Hanley et al., 2020; Kitson et al., 2020). As an illustration, flow is a self-transcendent experience elicited by the performance of a deeply rewarding yet appropriately challenging task, and its defining characteristic is a total attentional focus on the task alongside a correspondingly low level of self-consciousness (Nakamura and Csikszentmihalyi, 2002; Croom, 2015; Yaden et al., 2017). This complete absorption into an intrinsically positive state may lessen the weight of one’s negative affect. The self-transcendent experiences (e.g., flow, awe, compassion) derived from activities such as communal worship, musical performance, or immersion in vast scenes of nature have been linked to the heightening of positive affect, mitigation of negative affect, and efficacious therapeutic outcomes (e.g., Reynolds and Prior, 2006; Matthews and Cook, 2009; Moss, 2019; Kim et al., 2021; Monroy and Keltner, 2022).

As a caveat, profoundly positive as self-transcendent experiences may be, they do not aid the endurance of suffering solely by dint of being positive. Prior theories (e.g., Wong et al., 2021) make clear that the function of self-transcendence is not merely to reduce suffering, but also to engage with it in a manner that draws out some goodness despite its initial negativity. The positivity in self-transcendent experience does powerfully counterbalance the aversiveness of suffering, but there are additional benefits posed *via* bidirectional connections to the cognitive and motivational components of self-transcendence as well. These interactions will be discussed further in later sections.

## Effects of self-transcendent cognition on suffering

During moments of suffering, there is often a need to comprehend the causes and effects of the events that gave rise to the suffering (Gan et al., 2013, 2018; Calhoun, 2013a; Courtois, 2017; Park, 2020). This desire can be conceptualized as a need to reconcile the initial negative implications of suffering with one’s *global meaning*—fundamental beliefs of how the world works (e.g., the just world hypothesis or views of human nature) (Freh et al., 2013; Gan et al., 2013; Park and Gutierrez, 2013).

Two challenges may arise from such meaning-making processes. First, meaning-making involves the distressing possibility that one’s global meaning belief systems are too optimistic or otherwise unable to account for the meaning of a suffering event (e.g. Calhoun, 2013c; Pak, 2019; Frounfelker et al., 2020). In such instances, there appears to be an irreconcilable rupture in one’s conception of the world, and the failure to mend it may manifest in the intrusive thinking that produces the unexpected waves of grief mentioned prior (Calhoun, 2013b; Steger et al., 2015; McAdams and Jones, 2017; Pak, 2019; Milman et al., 2020). Second, to accommodate the meaning of suffering, individuals may shift their global meaning beliefs in a manner that makes negativity the predominant feature (Park et al., 2012; Gerrish et al., 2014; Gerrish and Bailey, 2020), as in the adoption of cynicism or existential nihilism. Such negative belief orientations are associated with poor well-being outcomes ranging from depressive and anxiety symptoms to suicidal ideation (Nierenberg et al., 1996; Dangel et al., 2018; Forsythe, 2021). Thus, the threat of failure to comprehend the meaning of a suffering event, as well as the potential negative global meaning one might derive, comprise a dimension of suffering that demands cognitive pathways of resolution.

Self-transcendent cognition—the reasoning and appraisal of phenomena according to a frame of reference beyond the self—gives rise to global meaning beliefs that may successfully account for suffering without setting negativity as the predominant feature. This self-transcendent cognition can occur *via* adopting a reasoning from the perspective of social entities beyond the self or even of abstract spiritual ones such as the idea of nature overall (e.g., Block, 2001; Frounfelker et al., 2020; Monroy and Keltner, 2022). Empirical research has found that people’s perceptions of meaning are greatly influenced by a sense of self-transcendence (e.g., making a positive impact on society) (Huang and Yang, 2022). As a concrete example, an examination of positive coping strategies in response to cancer found that reframing one’s terminal illness as part of natural cycles of “creation and destruction” may aid the peaceful acceptance of death (Block, 2001). Instead of prompting rumination over suffering as something happening specifically and unfairly to the self, the appraisal of one’s suffering as natural recontextualizes it as something shared with the whole of humanity. Moreover, such self-transcendent systems of global meaning may likewise successfully incorporate the suffering event—the prospect of death linked to “destruction” in the given example—into a perspective that also emphasizes positive features such as “creation,” thereby accounting for negativity in existence without sacrificing the positive. In contrast, cynicism or nihilism also do provide explanations for suffering, but they do not allow for the inclusion of such positivity and thereby lead to their associated negative well-being outcomes (Dangel et al., 2018; Forsythe, 2021). In providing an orientation of global meaning with a larger perspective and a focus on superordinate values, self-transcendent cognition may thus help resolve both the struggle to formulate acceptable meaning of suffering as well as the potential for maladaptive interpretations.

## Effects of self-transcendent motivation on suffering

On the level of motivation, the presence of suffering again provokes a twofold challenge. The first is that the inherent aversiveness of suffering triggers motivational systems oriented toward immediate alleviation of suffering (e.g., the desire to flee from the circumstances of one’s suffering). Though the hedonic motivation to mitigate displeasure is not necessarily maladaptive, fixation on such mitigation at the expense of other motives necessary for well-being may result in drives toward negative coping mechanisms such as avoidance, substance abuse, or self-escape (Carver and Connor-Smith, 2010; Freh et al., 2013; Giuntoli et al., 2021). Second, the aforementioned threats to cognitive global meaning may also hamper one’s eudaimonic motivations toward purpose (Park, 2008). Global meaning beliefs, in addition to providing coherent understandings of the world, also include prescriptive beliefs from which one may derive goal-oriented purpose in life (e.g., that one should help the sufferings of others) (Koltko-Rivera, 2004; Martela and Steger, 2016; McAdams and Jones, 2017; Park and Kennedy, 2017). To cast one’s global meaning beliefs into doubt may therefore produce the aimlessness and avolition characteristic of “languishing” (Keyes, 2002). The risk of succumbing to overly hedonistic motivations and the risk of falling to an outright vacuum of motivations are the two motivational challenges posed by suffering.

Self-transcendent motivation is defined here as the drive to be devoted to an entity beyond oneself in terms of one’s goals and purposes, which may lead to the tolerance and even embrace of suffering. For example, the desire among religious people to obey the edicts of their concept of the divine has been observed to compel outright embrace of one’s suffering as an opportunity to demonstrate religious devotion, even amid especially severe cases of pain such as cancer and childbirth (Krause and Bastida, 2011; Taghizdeh et al., 2017). The same motivation to willfully engage with one’s suffering in spite of its aversiveness has also been observed in non-religious forms of self-transcendence (e.g., desire to fulfill the self-sacrificial commitments of *agape* love) (Ozawa-de Silva et al., 2012; Van de Goor et al., 2020; Enright et al., 2022; Sørensen and Lien, 2022). As a point of differentiation, this motivation toward self-transcendence is fundamentally distinctive from the motivation toward self-escape (e.g., to “drown one’s sorrows” in alcohol): though both entail a desire to lessen the burden of self-awareness, self-transcendent motivation does so through affirming meaningful connections with entities beyond the self, whereas self-escape motivation is driven by a desire to numb meaningful thought and thereby avoid awareness of suffering (Baumeister, 1990; Yaden et al., 2017). Self-transcendent motivation, if adequately engaged-in, compels the individual beyond the push and pull of hedonistic sensualism toward the goal-oriented purposefulness characteristic of more holistic forms of well-being.

## Interactions between the three levels of self-transcendence

These three levels of self-transcendence do not operate in isolation from one another, but rather may each promote one another in the endurance of suffering. Past literature has suggested Meaning-Centered Coping wields its benefits along a holistic set of trajectories (e.g., positive reframing, engagement in meaningful activities, prosociality, etc.) (Wong, 2013; Eisenbeck et al., 2021), and we surmise the benefits of self-transcendence may function in a similar manner. We have established that the problem of suffering entails multiple fronts, including overwhelming negative affect and threats to systems of global meaning. This multifaceted nature of suffering ensures that the solutions best suited to combating it must be correspondingly holistic in their effects.

For example, self-transcendent experiences such as awe may give rise to a sense of meaning and provoke meaning-making processes that help solidify self-transcendent cognitive beliefs and values (Stellar et al., 2018; Zhao et al., 2019; Rivera et al., 2020; Monroy and Keltner, 2022; Kim et al., in press). Inversely, reframing one’s suffering through self-transcendent cognition may likewise induce some self-transcendent experiences, such as feelings of serenity derived from believing in natural forces greater than the self (Kreitzer et al., 2009; Garcia-Romeu, 2010). To contrast, some positive but non-self-transcendent experiences such as the pleasure of a good meal may alleviate the experience of suffering to some extent, but it would not address the needs for global meaning or purpose that have also been provoked by suffering.

Similarly, both self-transcendent experiences and self-transcendent cognitive beliefs can incentivize self-transcendent motivations. As an example, self-transcendent experiences from spending time in nature have been connected to a drive for prosocial behaviors due to feelings of greater connectedness with the world (Castelo et al., 2021). Likewise, the prescriptive values entailed in self-transcendent cognitive beliefs comprise the goals and purpose that compel self-transcendent motivations (Koltko-Rivera, 2004; McAdams and Jones, 2017). As an outcome of one’s cognitive religious beliefs, as noted prior, one may have a strong motivation to serve others (e.g., Krause and Bastida, 2011). In this fashion, all three levels of self-transcendence may feed into each other and thereby trigger cascades of each other’s mechanisms in aiding the endurance of suffering holistically.

## Downstream behavioral and social effects of self-transcendence

We conceptualize self-transcendence as a set of subjective mental processes, but its effects do not necessarily remain confined to the mind of the individual. Past research has suggested that subjective processes like meaning may stimulate constructive and prosocial behaviors (e.g., Van der Heyden et al., 2015; Klein, 2017; Eisenbeck et al., 2021), and we correspondingly hold that self-transcendent experiences may also have these positive effects on prosociality. For example, self-transcendent experiences such as awe motivate people to perform prosocial behaviors such as helping (Piff et al., 2015; Perlin and Li, 2020). In addition, self-transcendent experiences involving the sense of unity with others (e.g., communal worship, patriotic camaraderie in war) have been found to promote prosocial and pro-group behaviors (Swann and Buhrmester, 2015; Moss, 2019). These outward expressions of self-transcendence may also serve to signal prosociality to others and promote reciprocal positive social connections, giving rise to the systems of support that have been consistently observed as a potent resource for adaptive coping of suffering (Stallard et al., 2001; Matthews and Cook, 2009; Freh et al., 2013; Al-Kandari et al., 2017; Hoang et al., 2020; Fu et al., 2022).

## Conclusion

Our theoretical model maps out the precise effects self-transcendence may have in aiding the endurance of suffering. Prior research has measured self-transcendence primarily as general *personality traits* or *disposition* (e.g., Garcia-Romeu, 2010). Based on our theoretical model, it may be fruitful to adopt a different approach and measure self-transcendence as three interrelated *psychological processes*. Our approach provides the theoretical basis for operationalizing self-transcendence empirically at different levels of analysis. Past work has referenced self-transcendence as a potential source of many factors that help people endure suffering (e.g., meaning, problem-focused coping) (Frankl, 1946/1992; Matthews and Cook, 2009; Eisenbeck et al., 2021; Sanchez-Ruiz et al., 2021; Avsec et al., 2022; Eisenbeck et al., 2022). By specifying the affective, cognitive, and motivational mechanisms through which self-transcendence helps the endurance of suffering, our work sharpens the precision of past theoretical models and allows us to more effectively assess the beneficial effects of self-transcendence through quantitative and qualitative research. The story of Gilgamesh (*ca.* 2,750–2,500 B.C.E./2003) culminates in the king attaining a level of peace upon looking out over his city walls and recognizing his contribution to the happiness of his people. With our theoretical framework for the functions of self-transcendence, we hope to elucidate how the people of modernity may likewise endure and transcend the inevitable suffering in life by transcending the self.

## Data availability statement

The original contributions presented in the study are included in the article/supplementary material, further inquiries can be directed to the corresponding authors.

## Author contributions

BG and FY conceived the ideas and revised the manuscript. BG wrote the initial draft. All authors contributed to the article and approved the submitted version.

## Conflict of interest

The authors declare that the research was conducted in the absence of any commercial or financial relationships that could be construed as a potential conflict of interest.

## Publisher’s note

All claims expressed in this article are solely those of the authors and do not necessarily represent those of their affiliated organizations, or those of the publisher, the editors and the reviewers. Any product that may be evaluated in this article, or claim that may be made by its manufacturer, is not guaranteed or endorsed by the publisher.
